# Exploratory Graph Analysis Failed to Reproduce the Hypothesized Functional Zoning Framework in the OECD Survey on Social and Emotional Skills

**DOI:** 10.1002/pchj.70108

**Published:** 2026-06-17

**Authors:** Lidan Wang, Bo Ning

**Affiliations:** ^1^ Research Institute for International and Comparative Education & Lab for Educational Big Data and Policymaking, Shanghai Normal University Shanghai China

**Keywords:** big five personality traits theory, cultural variations, exploratory graph analysis, functional zoning framework, survey on social and emotional skills

## Abstract

The OECD's first Survey on Social and Emotional Skills (SSES) in 2019 assessed 10‐ and 15‐year‐olds across ten cities, employing a framework of 15 skills organized into five dimensions aligned with the Big Five personality traits. While this adult‐derived model offers a parsimonious structure for measuring social–emotional skills, its applicability to children and early adolescents across diverse cultural contexts remains largely untested. This study examined the structural validity of the SSES framework employing exploratory graph analysis (EGA)—a network psychometrics technique using skill facets as nodes, regularized partial correlations as edge weights, and the walktrap algorithm for community detection. Drawing on self‐report data from 60,440 participants aged 10 and 15 across ten international cities, our analysis revealed that contrary to expectation, the hypothesized five‐factor structure did not emerge in any age group or city. Among 10‐year‐olds, skills remained largely undifferentiated or showed only preliminary clustering in most cities, suggesting developmental immaturity in functional organization. Among 15‐year‐olds, three to four distinct dimensions emerged, with significant cross‐city variation in composition. Developmental trajectories also differed culturally: Houston, Sintra, and Istanbul exhibited early‐but‐stagnant differentiation, while Suzhou showed rapid progression from undifferentiated to four‐dimensional structure. These findings identify key limitations in applying the SSES framework: (1) the pattern of conditional dependencies among skill facets violates the local independence assumption underlying the five‐factor model; (2) the organization of skills is not equivalent across ages: 10‐year‐olds show largely undifferentiated structures while 15‐year‐olds exhibit clearer differentiation; and (3) dimensional configurations vary substantially across cities, challenging assumptions of cross‐cultural universality. The results inform researchers and policymakers about the framework's limitations and highlight the need for age‐ and culture‐sensitive approaches to social–emotional skills assessment.

## 1 Introduction

### 1.1 Operationalizing Social–Emotional Skills Through the Lens of the Big Five Personality Traits

Social–emotional skills (SES), also known as social and emotional competencies, refer to essential skills that enable individuals to effectively navigate the complexities of their emotions and social interactions (Osher et al. 2016; Schoon 2021). Previous research has indicated that social–emotional skills are crucial for students' academic performance (Guo et al. 2023; McCormick et al. 2021; Schoon 2021) and their physical and mental health (Anglim et al. 2020; Strickhouser et al. 2017). Researchers have developed several theoretical frameworks to measure social–emotional skills (Lee and Junus 2024).

In 2019, the Organization for Economic Co‐operation and Development (OECD) initiated their first Survey on Social and Emotional Skills (SSES) (Steponavičius et al. 2023). The functional zoning framework of the SSES operationalizes the Big Five Personality Traits Theory (the Big Five) through 15 specific skill facets. The five hypothesized dimensions are: Task Performance (conscientiousness), encompassing Responsibility, Persistence, and Self‐Control; Emotion Regulation (emotional stability), encompassing Stress Resistance, Optimism, and Emotional Control; Collaboration (agreeableness), encompassing Empathy, Trust, and Cooperation; Open‐Mindedness (openness to experience), encompassing Curiosity, Tolerance, and Creativity; and Engaging with Others (extraversion), encompassing Sociability, Assertiveness, and Energy (Kankaraš and Suarez‐Alvarez 2019). This “functional zoning” hypothesis posits that these 15 skills naturally cluster into five distinct but interrelated dimensions, mirroring the organization of adult personality traits (Goldberg 1993).

### 1.2 Theoretical and Empirical Challenges in Big Five‐Based Social–Emotional Research

The application of an adult‐derived model to children and adolescents raises fundamental developmental questions. A recent study based on the OECD SSES 2019, examining 10‐ and 15‐year‐olds, suggests that the social–emotional skills of early adolescents form denser and more integrated network structures, suggesting a reorganization of skills from childhood to early adolescence (Huo and Ning 2025). Neurodevelopmental research supports this view: the adolescent brain undergoes extensive reorganization characterized by both functional segregation (specialization of distinct regions) and functional integration (long‐range connectivity between regions) (Tononi et al. 1994; Stevens 2016). If social–emotional skills follow similar developmental principles, we would expect 10‐year‐olds to exhibit less differentiated skill structures than 15‐year‐olds, with functional zones emerging progressively with age. The SSES framework's assumption of a stable five‐dimension structure across these ages may therefore overlook critical developmental transformations.

Beyond developmental considerations, the cross‐cultural validity of the Big Five‐based framework warrants careful examination. Cultural psychologists have long argued that personality and social–emotional functioning are shaped by prevailing cultural values and socialization practices (Markus and Kitayama 1991; Triandis 1995). Of particular relevance is the dimension of individualism–collectivism (Hofstede 2001). Individualistic societies (e.g., North American and Western European contexts) emphasize personal autonomy, self‐expression, and individual achievement, potentially fostering clearer differentiation of self‐oriented skills (e.g., Task Performance, Assertiveness). Collectivist societies (e.g., East Asian contexts) prioritize group harmony, interpersonal connectedness, and role‐based obligations, potentially leading to greater integration of relational skills (e.g., Collaboration, Empathy) and different patterns of skill organization (Markus and Kitayama 1991; Heine et al. 1999). The ten SSES 2019 participating cities span diverse cultural contexts: Suzhou (China) represents a Confucian‐heritage, collectivist culture; Daegu (Korea) similarly reflects East Asian collectivism; Istanbul (Turkey) embodies a unique intersection of Eastern and Western influences; Houston (US) and Ottawa (Canada) represent Western individualistic contexts; while Helsinki (Finland), Bogota and Manizales (Colombia), Moscow (Russia), and Sintra (Portugal) offer additional cultural variation. Examining how the SSES structure performs across these diverse settings provides a rigorous test of its cross‐cultural generalizability.

Establishing the structural validity of the SSES framework is not merely an academic exercise; it has significant practical implications. The SSES has already informed education policy in multiple countries (OECD 2021), with findings from its framework being used to guide curriculum development, resource allocation, and intervention design (Steponavičius et al. 2023). If the assumed five‐dimension structure does not hold across age groups or cultural contexts, several consequences follow: (1) cross‐age comparisons (e.g., tracking skill development from age 10 to 15) may be invalid if the meaning of the dimensions differs across ages; (2) cross‐city comparisons (e.g., ranking cities on specific dimensions) may be misleading if the dimensions are not culturally equivalent; and (3) interventions targeting specific dimensions (e.g., a program to improve Emotion Regulation) may be misdirected if the targeted skills do not cohere as hypothesized in the local context. Thus, evaluating the SSES framework's structural invariance is essential for ensuring that subsequent research and policy applications rest on a solid empirical foundation. This study addresses this gap by systematically examining the emergent organization of social–emotional skills across ten cities and two age groups, using a data‐driven approach that allows structures to vary naturally rather than imposing a pre‐specified model.

### 1.3 Emerging Evidence Questioning the Applicability of the Big Five Framework to Social–Emotional Skills

Recently, Shetty et al.'s (2023) team conducted an investigation to explore the factor structure of personality by analyzing the Big Five personality factors within a sample population from Karnataka, South India. Their Factor Analysis revealed that none of the identified factors fit into the original five factors, raising questions about the cross‐cultural validity of the Big Five model. Not coincidentally, Laajaj et al. (2019) adopted a comprehensive psychometric approach to analyze 29 face‐to‐face surveys involving 94,751 respondents across 23 low‐ and middle‐income countries. Their findings demonstrated that commonly used personality questions often fail to accurately measure the intended traits and exhibit low validity.

Still, the differences between social–emotional skills and personality traits should be cautious concerning the plasticity of the former and the stability of the latter. The Big Five describes personality traits that are generally believed to be inherent and relatively consistent over time (Steponavičius et al. 2023). While social–emotional skills are more dynamic and can be improved over time. Specifically, a person may act in a certain way but possess the ability to behave differently when the situation requires it (Soto et al. 2022; Steponavičius et al. 2023). These characteristics make SSES susceptible to cultural and individual development and therefore require consideration of cross‐cultural as well as age‐validity. In line with the aforementioned findings, the OECD SSES Technical Report revealed non‐invariance measurement across the ten participating cities, highlighting the need for caution when making cross‐site or between‐age comparisons of the survey results (Lee and Junus 2024; OECD 2021).

### 1.4 The Present Study

The present study aims to investigate the structure of social–emotional skills among 10‐year‐olds and 15‐year‐olds in ten cities, using data from the first wave of OECD SSES. Exploratory graph analysis (EGA), a new data‐driven technique within the framework of network psychometrics, was employed to identify the underlying dimensions of social–emotional skills and to assess their alignment with the hypothesized five dimensions based on the Big Five. To the best of our knowledge, this study is the first to evaluate the OECD SSES measurement framework, which has been widely used in many publications.

## 2 Methods

### 2.1 Sample and Measures

The OECD SSES was an international assessment conducted in 2018–2019, launched to assess social–emotional competence among 10‐ and 15‐year‐old students. The sample consisted of 60,440 participants from ten cities across nine countries. Among them, 49.7% of the 10‐year‐olds and 51.6% of the 15‐year‐olds were female. The sample sizes for the 10‐year‐old cohorts varied widely across the ten cities, with figures ranging from 2191 in Sintra to 3631 in Suzhou. Similarly, for the 15‐year‐olds, Sintra had the smallest group with 1633 participants, whereas Suzhou had the largest one with 3611 participants. All cities exhibited a balanced gender representation. Table 1 displays the demographic characteristics of the study sample across the ten cities.

**TABLE 1:** Demographic characteristics of the sample across cities.

City	Age	*N*	Gender (female %)
1. Ottawa, Canada	10	3159	50.3
	15	2173	49.6
2. Houston, US	10	3308	50.7
	15	3092	52.6
3. Bogota, Colombia	10	3400	50.8
	15	3354	49.8
4. Manizales, Colombia	10	3218	50.9
	15	3531	52.0
5. Helsinki, Finland	10	2992	50.1
	15	2433	52.8
6. Moscow, Russia	10	3336	48.4
	15	3417	49.0
7. Istanbul, Turkey	10	2697	49.9
	15	3166	58.7
8. Daegu, Korea	10	3006	49.3
	15	3092	51.2
9. Sintra, Portugal	10	2191	52.7
	15	1633	52.4
10. Suzhou, China	10	3631	45.5
	15	3611	48.7
Total	10	30,938	49.7
	15	29,502	51.6

This study used self‐report data based on student questionnaires from 2019 SSES. As mentioned above, the OECD constructed the social–emotional skills assessment framework based on the Big Five Personality Traits Theory, which consisted of five dimensions. Within each dimension were three skill facets, and these were assessed using eight items on a 5‐point Likert scale (see Table 2 for details). All city samples showed good reliability for these scales, as detailed in the OECD Technical Report (OECD 2021).

**TABLE 2:** Dimensions and facets of skills in the OECD social–emotional skills framework.

Dimensions	Facets	Description	Sample items
Task performance (Conscientiousness)	Responsibility (RES)	Able to honour commitments, and be punctual and reliable	I keep promise
	Self‐Control (SEL)	Able to avoid distractions and sudden impulses and focus attention on the current task in order to achieve personal goals	I avoid mistakes by working carefully
	Persistence (PER)	Persevering in tasks and activities until they get done	I keep working on a task until it is finished
Emotion regulation (Emotional stability)	Stress Resistance (STR)	Effectiveness in modulating anxiety and able to calmly solve problems (is relaxed, handles stress well)	I am relaxed and handle stress well
	Optimism (OPT)	Positive and optimistic expectations for self and life in general	I believe good things will happen to me
	Emotional Control (EMO)	Effective strategies for regulating temper, anger and irritation in the face of frustrations.	I stay calm even in tense situations
Collaboration (agreeableness)	Empathy (EMP)	Understanding and caring for others and their well‐being that leads to valuing and investing in close relationships	I understand what others want
	Trust (TRU)	Assuming that others generally have good intentions and forgiving those who have done wrong	I believe that most people are honest
	Cooperation (COO)	Living in harmony with others and valuing interconnectedness among all people	I get along well with others
Open‐mindedness (openness to experience)	Curiosity (CUR)	Interest in ideas and love of learning, understanding and intellectual exploration; an inquisitive mind‐set	I love learning new things in school
	Tolerance (TOL)	Is open to different points of view, values diversity, is appreciative of foreign people and cultures	I like hearing about other cultures and religions
	Creativity (CRE)	Generating novel ways to do or think about things through exploring, learning from failure, insight and vision	I sometimes find a solution that other people don't see
Engaging with others (Extraversion)	Sociability (SOC)	Able to approach others, both friends and strangers, initiating and maintaining social connections	I like to be with friends
	Assertiveness (ASS)	Able to confidently voice opinions, needs, and feelings, and exert social influence	I like being a leader in my class
	Energy (ENE)	Approaching daily life with energy, excitement and spontaneity	I maintain high energy throughout the day

### 2.2 Data Analysis

#### 2.2.1 Dimensionality Assessment Using Exploratory Graph Analysis (EGA)

This study employed exploratory graph analysis (EGA) to examine the dimensional structure of social–emotional skills. EGA is a network psychometrics technique that estimates the number of latent dimensions underlying multivariate data by identifying clusters of highly interconnected variables (Golino and Epskamp 2017). Unlike traditional factor analysis, which forces items onto pre‐specified or statistically derived latent factors under the assumption of local independence, EGA takes a data‐driven approach that allows the emergent organization of skills to be revealed directly from the pattern of associations among observed variables. This characteristic makes EGA particularly suitable for exploring how functional zones might develop and differ across age groups and cultural contexts, as it does not assume that the same dimensional structure applies universally. The EGA procedure followed a step‐by‐step workflow.

Step 1: Network estimation. For each city and age group separately, we estimated a network of associations among the 15 skill facets. Nodes represented the skill facets (e.g., Responsibility, Empathy). Edge weights were calculated as regularized partial correlations using the graphical least absolute shrinkage and selection operator (GLASSO) with an extended Bayesian information criterion (EBIC) hyperparameter (*γ*) set to 0.5, as recommended for psychological data to balance sensitivity and specificity (Golino and Epskamp 2017; Epskamp and Fried 2018). This regularization technique shrinks small correlations to zero, producing a sparse network that highlights the most robust associations while reducing false positives. The EBIC tuning parameter was tested across 100 values to ensure optimal model selection.

Step 2: Community detection. The walktrap algorithm (Pons and Latapy 2006) was applied to the regularized partial correlation matrix to identify communities—clusters of nodes that are more densely connected to each other than to nodes in other clusters. The algorithm operates by simulating random walks on the network; nodes that are frequently visited in sequence are considered part of the same community. The number of communities was determined empirically without pre‐specification, allowing the data to reveal the emergent dimensional structure. Communities were interpreted as latent dimensions of social–emotional skills, consistent with network psychometrics conventions (Golino et al. 2020).

Step 3: Visualization and interpretation. EGA produces a network plot that visualizes the identified communities using node colors and edge thickness to represent connection strength. This visualization indicates not only the number of dimensions to retain, but also which skill facets cluster together and their levels of association (Golino et al. 2020). For this study, we examined the community solutions across all ten cities and two age groups to assess: (a) whether the hypothesized five dimensions emerged; (b) how dimensional structures varied by age and city; and (c) whether consistent patterns of skill organization could be identified.

**FIGURE 1:**
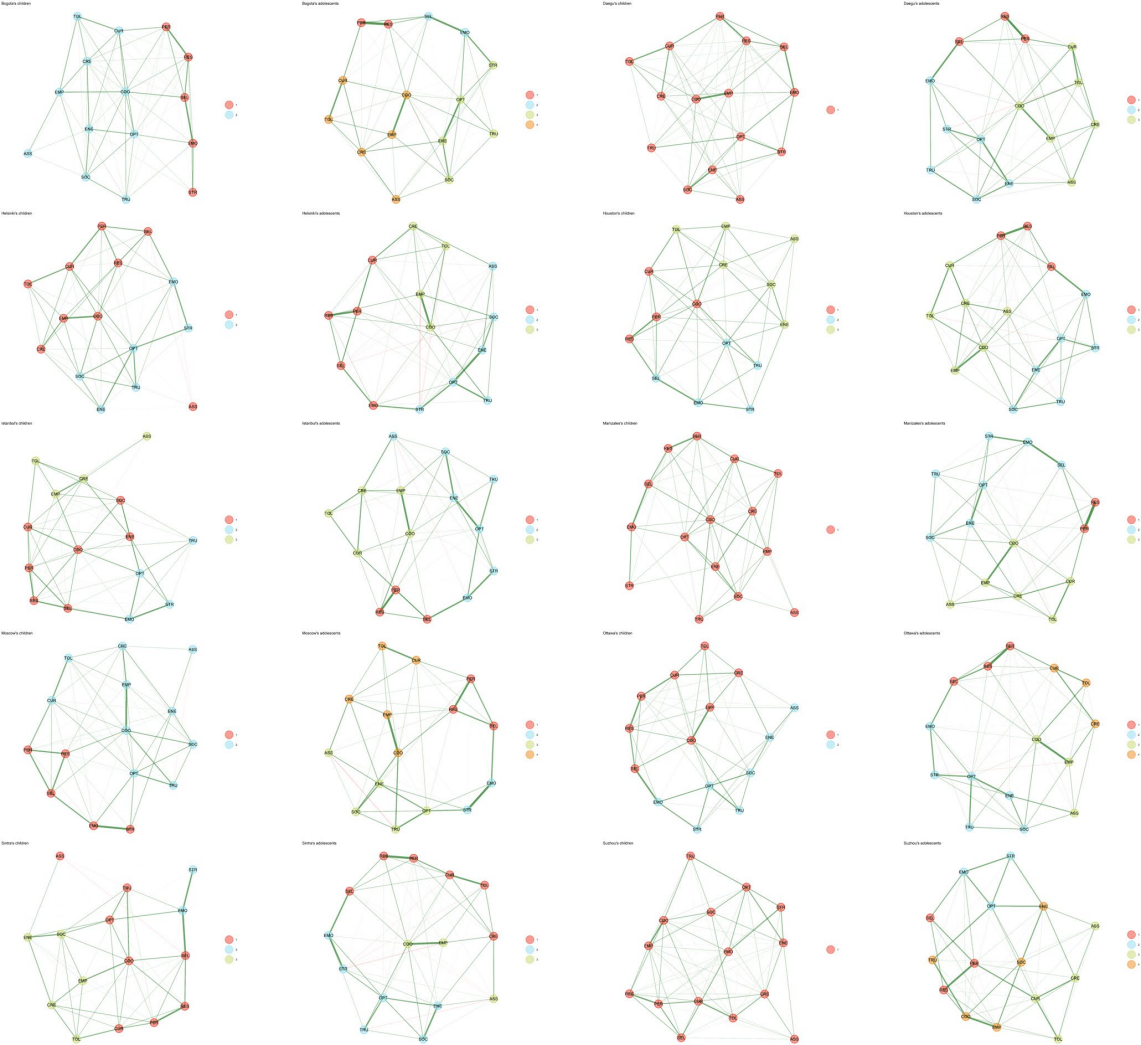
Graph of the functional zones of social–emotional skills in each city and age cohort.

To ensure clarity in interpreting age‐related differences, we operationally defined “undifferentiated” structures as solutions where all 15 skill facets formed a single community (i.e., modularity *Q* < 0.25; Newman and Girvan 2004), indicating insufficient segregation to identify distinct functional zones. “Preliminary differentiation” referred to solutions with 2–3 communities, suggesting incomplete functional segregation. “Differentiated” structures referred to solutions with 4–5 communities showing clear separation and robust within‐cluster connections. Figure 1 shows the emerging functional zones of social–emotional skills in each city and age cohort.

Software and packages. All analyses were performed using R version 4.4.1 in R Studio 2024.04.2‐764, with the following R packages: EGAnet (version 2.0.7) for exploratory graph analysis (Christensen and Golino 2024); qgraph (version 1.9.8) for network visualization (Epskamp et al. 2012); and igraph (version 2.0.3) for community detection algorithms (Csárdi and Nepusz 2006).

#### 2.2.2 Sensitivity Analysis and Bootstrap EGA for Stability

In order to evaluate the robustness of the EGA results, we conducted two complementary stability analyses. First, we performed a sensitivity analysis by varying the hyperparameter *γ* (gamma) in the extended Bayesian information criterion (EBIC) used for model selection in the graphical lasso (Epskamp and Fried 2018). Specifically, we re‐estimated the regularized partial correlation networks with *γ* values from 0 to 1 in steps of 0.25, which range from a more exploratory to a more conservative penalty, with *γ* = 0.5 to be the recommended value (Foygel and Drton 2010).

Second, we applied bootstrap exploratory graph analysis (bootEGA), a resampling‐based method that combines exploratory graph analysis with bootstrapping (Christensen and Golino 2021). BootEGA repeatedly estimates the network structure on bootstrap samples to compute measures of structural consistency and dimension stability. Descriptive statistics, especially median number of dimensions found across the bootstraps (median. dim) was used to exam the stability (Christensen and Golino 2021). Together, these two approaches provide a comprehensive assessment of the robustness of our network estimates against both hyperparameter selection and sampling variability.

## 3 Results

### 3.1 EGA‐Retrieved Dimensional Structure

The functional zoning of social–emotional skills is a key indicator of their maturity level. Tables 3 and 4 illustrate the dimensions of social–emotional skills identified in each city among 10‐year‐olds and 15‐year‐olds. Generally, the two age groups exhibit very distinct clustering, and no city in each age group fits the five dimensions of the SSES announced by OECD.

**TABLE 3:** Network structure of social‐emotional skills measurement model of children.

Dimensions	Facets	Suzhou	Daegu	Manizales	Bogota	Moscow	Ottawa	Helsinki	Houston	Sintra	Istanbul
Task Performance	Responsibility (RES)	RES	RES	RES	RES	RES	RES	RES	RES	RES	RES
	Persistence (PER)	PER	PER	PER	PER	PER	PER	PER	PER	PER	PER
	Self‐Control (SEL)	SEL	SEL	SEL	SEL	SEL	SEL	SEL	COO	SEL	SEL
Emotion Regulation	Emotional Control (EMO)	EMO	EMO	EMO	EMO	EMO	CUR	CUR	CUR	CUR	CUR
	Optimism (OPT)	OPT	OPT	OPT	STR	STR	TOL	TOL	OPT	OPT	COO
	Stress Resistance (STR)	STR	STR	STR	OPT	OPT	CRE	CRE	STR	COO	SOC
Collaboration	Trust (TRU)	TRU	TRU	TRU	TRU	TRU	COO	ASS	EMO	TRU	ENE
	Cooperation (COO)	COO	COO	COO	COO	COO	EMP	COO	TRU	ASS	ASS
	Empathy (EMP)	EMP	EMP	EMP	EMP	EMP	TRU	EMP	SEL	EMO	EMP
Open‐Mindedness	Curiosity (CUR)	CUR	CUR	CUR	CUR	CUR	EMO	EMO	EMP	STR	TOL
	Tolerance (TOL)	TOL	TOL	TOL	TOL	TOL	OPT	OPT	TOL	TOL	CRE
	Creativity (CRE)	CRE	CRE	CRE	CRE	CRE	STR	STR	CRE	CRE	EMO
Engaging with Others	Sociability (SOC)	SOC	SOC	SOC	SOC	SOC	SOC	SOC	SOC	SOC	OPT
	Energy (ENE)	ENE	ENE	ENE	ENE	ENE	ENE	ENE	ENE	ENE	STR
	Assertiveness (ASS)	ASS	ASS	ASS	ASS	ASS	ASS	TRU	ASS	EMP	TRU

*Note:* Skills in the same dimension are in the same color.

**TABLE 4:** Network structure of social–emotional skills measurement model of adolescents.

Dimensions	Facets	Manizales	Daegu	Houston	Istanbul	Helsinki	Sintra	Bogota	Moscow	Suzhou	Ottawa
Task performance	Responsibility (RES)	RES	RES	RES	RES	RES	RES	RES	RES	RES	RES
	Persistence (PER)	PER	PER	PER	PER	PER	PER	PER	PER	PER	PER
	Self‐Control (SEL)	SEL	SEL	SEL	SEL	SEL	SEL	SEL	SEL	SEL	SEL
Emotion regulation	Emotional Control (EMO)	EMO	EMO	EMO	EMO	EMO	CUR	EMO	EMO	EMO	EMO
	Optimism (OPT)	OPT	OPT	OPT	OPT	CUR	TOL	OPT	STR	OPT	OPT
	Stress Resistance (STR)	STR	STR	STR	STR	OPT	CRE	STR	OPT	STR	STR
Collaboration	Trust (TRU)	TRU	TRU	TRU	TRU	STR	ASS	TRU	TRU	TRU	TRU
	Cooperation (COO)	SOC	SOC	SOC	SOC	TRU	COO	SOC	SOC	COO	SOC
	Empathy (EMP)	ENE	ENE	ENE	ENE	SOC	EMP	ENE	ENE	EMP	ENE
Open‐Mindedness	Curiosity (CUR)	CUR	CUR	CUR	ASS	ENE	EMO	CUR	ASS	SOC	CUR
	Tolerance (TOL)	TOL	TOL	TOL	CUR	ASS	OPT	TOL	CUR	ENE	TOL
	Creativity (CRE)	CRE	CRE	CRE	TOL	EMP	STR	CRE	TOL	CUR	CRE
Engaging with others	Sociability (SOC)	COO	COO	COO	CRE	COO	SOC	COO	CRE	TOL	COO
	Energy (ENE)	EMP	EMP	EMP	COO	TOL	ENE	EMP	COO	CRE	EMP
	Assertiveness (ASS)	ASS	ASS	ASS	EMP	CRE	TRU	ASS	EMP	ASS	ASS

*Note:* Skills in the same dimension are in the same color.

For the 10‐year‐olds, the structure of the social–emotional skills is in an undifferentiated or preliminary differentiation status in most cities (see Table 3). In Suzhou, Daegu, and Manizales, no distinct dimensions emerged from our EGA modelling. The 15 skill facets in these sites formed a single connected component with modularity below 0.25 (Newman and Girvan 2004), indicating weak segregation between hypothesized dimensions. In Bogota, Moscow, Ottawa, and Helsinki, two dimensions emerged. In these sites, Task Performance and Emotion Regulation were closely linked, while Open‐Mindedness and Engaging with Others formed another distinct cluster. Collaboration skills consistently aligned with one of these two groups. The emergence of two communities, along with overlapping dimensions, points to incomplete functional segregation. Notably, Bogota and Moscow exhibited the same dimensional structure, with Collaboration linking with Open‐Mindedness and Engaging with Others. Ottawa and Helsinki shared a similar structure, where Collaboration skills were primarily linked with Task Performance and Emotion Regulation. In Houston, Sintra, and Istanbul, three dimensions emerged from the modeling. In these sites, Task Performance was linked with some Emotion Regulation skills to form one dimension, while Engaging with Others was linked with some Open‐Mindedness skills to form another dimension. The third dimension consisted primarily of Collaboration skills, alongside some skills from Emotion Regulation or Engaging with Others.

Compared to 10‐year‐old students, 15‐year‐old students show a significant improvement in the functional zoning of social–emotional skills in general (see Table 4). There were three or four dimensions emerging in the EGA modeling in each city. Three dimensions were identified in Manizales, Daegu, Houston, Istanbul, Helsinki, and Sintra. Generally, skills from Task Performance separated as one dimension in most cities, skills from Emotion Regulation and Collaboration merged as the second dimension, and skills from Open‐Mindedness and Engaging with Others merged as the third dimension. The four‐dimension structures were identified in four cities and in diverse combinations. These cities include Suzhou, Bogota, Ottawa, and Moscow. Generally, Task Performance skills emerged as one dimension in most cities; the skills of Engaging with Others are separated or merged with those of Open‐Mindedness, and the skills of Collaboration are merged with those of Emotion Regulation or Open‐Mindedness. Table 5 illuminates the number of functional zones and dominant cluster compositions by city and age cohort.

**TABLE 5:** Number of functional zones and dominant cluster compositions by city and age cohort.

City	10‐year‐olds		15‐year‐olds	
	Number of dimensions	Dominant clusters	Number of dimensions	Dominant clusters
Suzhou	1	Undifferentiated	4	TP\|ER\|COL (EWO mixed)\|OPE (EWO mixed)
Daegu	1	Undifferentiated	3	TP\|ER + Mixed\|OPE + Mixed
Manizales	1	Undifferentiated	3	TP (part)\|ER + Mixed \| OPE + Mixed
Bogota	2	TP (ER mixed)\|COL+OPE + EWO (ER mixed)	4	TP (part)\|Mixed\|Mixed\|OPE + Mixed
Moscow	2	TP (ER mixed)\|COL+OPE + EWO (ER mixed)	4	TP\|ER (part)\|EWO + Mixed\|OPE (COL mixed)
Ottawa	2	TP + OPE (COL mixed)\|ER + EWO (COL mixed)	4	TP\|ER + Mixed\|OPE\|Mixed
Helsinki	2	TP + OPE + Mixed\|ER + EWO + Mixed	3	TP + Mixed\|EWO + Mixed\| + Mixed
Houston	3	Mixed\|ER + Mixed\|EWO + Mixed	3	TP\|ER + Mixed\|OPE + Mixed
Sintra	3	TP + Mixed\|ER (part)\|EWO (OPE mixed)	3	TP + OPE\|Mixed\|ER + Mixed
Istanbul	3	TP + Mixed\|Mixed\|ER (COL mixed)	3	TP\|ER + EWO (COL mixed)\|OPE (COL mixed)

*Note:* “Mixed” indicates that the skills included do not form a complete SSES dimension and come from more than one dimension. “part” means that only two skills from that dimension are included. “+” indicates merged dimensions; “\|” separates distinct dimensions.

Abbreviations: COL, collaboration; ER, emotion regulation; EWO, engaging with others; OPE, open‐mindedness; TP, task performance.

The sequential ordering of dimensions (Task Performance → Emotion Regulation → Collaboration → Open‐Mindedness → Engaging with Others) was derived from network‐based distance metrics. Specifically, we computed the average shortest path length between skill facets belonging to different hypothesized dimensions using the walktrap‐derived community structure. For each pair of dimensions, we calculated the mean of all pairwise distances (inverse of regularized partial correlations) between their constituent facets, then normalized these distances to a 0–1 scale. Dimensions with the smallest average distances were considered adjacent in the continuum; those with the largest average distances were considered poles. This approach revealed that Task Performance and Engaging with Others consistently showed the greatest separation across all cities and age groups, with the other three dimensions occupying intermediate positions. The arrow notation (→) indicates increasing distance from Task Performance, not causal direction or developmental sequence, though the ordering aligns with the conceptual structure of the Big Five (Goldberg 1993).

### 3.2 Robustness Checks of the EGA Solutions

We first performed a sensitivity analysis by varying the EBIC hyperparameter *γ* (0, 0.25, 0.5, 0.75, 1). For 19 out of 20 city‐age groups, the number of dimensions at *γ* = 0.5 was identical to the original EGA result (Table S1). The only exception was Manizales10, where *γ* = 0.5 yielded two dimensions while the original EGA gave one dimension. We then conducted bootEGA with 1000 non‐parametric resamples to assess stability against sampling variability. The descriptive statistics revealed that the median bootEGA dimension matched the original EGA solution in 16 out of 20 groups (80%). The four mismatched cases were Ottawa15, Bogota15, Manizales10, and Helsinki10. Taken together, the sensitivity and bootstrap analyses confirm that the dimensional structures are robust for the vast majority of city‐age groups, and the few inconsistencies occur in developmentally or culturally sensitive cases.

To assess the potential hierarchical structure of the OECD model and to test whether the failure of EGA is method‐specific, we used skill‐level scores as indicators for each domain and conducted hierarchical confirmatory factor analysis (CFA) and bifactor CFA, respectively. Similarly, the results imply that the five‐domain framework of the OECD may not be an accurate categorization of the 15 skill facets into five domains, regardless of whether a hierarchical or a bifactor structure is assumed. Detailed results are provided in the *Hierarchical and Bifactor CFA Results* section of the Supporting Information.

## 4 Discussion

Based on exploratory graph analyses and the OECD 2019 Survey on Social and Emotional Skills, this study uncovered and compared the underlying functional zones of social–emotional skills among 10‐year‐olds and 15‐year‐olds across ten cities. The findings challenge their theoretical hypothesis of a five‐dimension construct of social–emotional skills based on the Big Five Personality Traits Theory and suggest that the functional zones of the social–emotional skills are largely different across cities and age groups.

### 4.1 Developmental Differentiation: Integration With Neurodevelopmental Evidence

A body of literature has shown that there is an inextricable relationship between social emotions (e.g., empathy) and the human brain (Maliske and Kanske 2022; Zaki and Ochsner 2012). The human brain is organized based on two fundamental principles: functional integration through long‐range connections and functional segregation into distinct regions through local differentiation (Tononi et al. 1994). Research in neuroscience suggested that the development of social–emotional skills is not only about the improvement of individual skills but also the increase of interconnections among skills and the emergence of functional zones that group related skills (Harris et al. 2022). The current study confirmed that the social–emotional skills of the 10‐year‐olds are in a state of holism, with no or raw differentiation emerging in the ten participating cities. Among the 15‐year‐olds, however, three or four functional zones of social–emotional skills emerged in most cities.

Our findings suggest that social–emotional skills follow a parallel developmental trajectory. The undifferentiated structures in Suzhou, Daegu, and Manizales at age 10, where all skill facets formed a single interconnected network, may reflect the immature state where distinct competencies have not yet coalesced into specialized functional systems. By age 15, the emergence of 3–4 distinct dimensions in all ten cities suggests that functional specialization has progressed, though full five‐dimension differentiation (as in adults) may not be achieved until later adolescence or early adulthood. This interpretation aligns with Harris et al.'s (2022) Social Emotional Ability Development (SEAD) model, which posits that emotional competencies become increasingly differentiated and integrated with age through reciprocal interactions between developing neural systems and social experiences.

The observed developmental progression from undifferentiated skill structures at age 10 to clearer functional differentiation at age 15 aligns systematically with Piaget's cognitive stage theory. According to Piaget, children aged 10 years typically operate within the concrete operational stage (approximately ages 7–11), characterized by logical reasoning that remains tied to concrete objects and specific contexts (Piaget and Inhelder 1969). During this stage, children's cognitive structures are organized in a relatively integrated, holistic manner. They can perform mental operations on tangible problems but have not yet developed the capacity for abstract, hypothetical, or systematic reasoning (Huitt and Hummel 2003; Piaget and Inhelder 1969). By contrast, most 15‐year‐olds have entered the formal operational stage (approximately ages 11–16), marked by the capacity for abstract, hypothetical, and systematic thinking, which involves progressive functional segregation—the ability to differentiate and coordinate multiple dimensions of a problem simultaneously (Piaget and Inhelder 1969).

The observation that even at age 15, no city showed the full five‐dimension structure has important implications. It suggests that the adult‐derived Big Five model may represent an endpoint of development that is not yet reached by early adolescence. Longitudinal research tracking individuals from childhood through early adulthood would be valuable to map the full developmental trajectory of skill differentiation and determine when and under what conditions the five‐dimension structure emerges.

### 4.2 Cross‐Cultural Variation: Toward Context‐Sensitive Explanations

The observed cross‐city differences in dimensional structures invite cautious interpretation rooted in cultural and educational contexts.

Suzhou's rapid development. Suzhou's rapid progression from undifferentiated to four‐dimensional structure aligns with China's recent policy emphasis on holistic social–emotional learning in compulsory education (Liu 2025) and high academic expectations that may accelerate skill differentiation in Task Performance and Emotion Regulation. Chinese middle schools, in particular, place a strong emphasis on academic achievement and self‐discipline (Li 2012). Moreover, this developmental pattern aligns with Confucian‐heritage values that prioritize self‐cultivation, perseverance, and emotional moderation (Kim and Park 2006), suggesting that cultural frameworks may also shape the pace and form of skill organization during adolescence.

Early‐but‐stagnant development in Houston, Sintra, and Istanbul. These three cities showed three dimensions at both ages, suggesting earlier onset of differentiation but limited subsequent progression. Houston's pattern may reflect the influence of universal Social and Emotional Learning (SEL) mandates in many U.S. school districts (CASEL 2020), potentially accelerating early skill organization. However, the lack of further differentiation by age 15 raises questions: Do these educational programs emphasize integration over specialization? Or do cultural factors in these contexts, perhaps greater emphasis on relational skills (Collaboration) over individual achievement dimensions, produce a different developmental endpoint? Sintra (Portugal) and Istanbul (Turkey) represent Southern European and transcontinental contexts where collectivist orientations may prioritize social harmony over individual skill differentiation (Hofstede 2001), potentially explaining why Collaboration remained merged with other dimensions rather than separating independently.

Helsinki's moderate development. Finland's educational system is renowned for its emphasis on student well‐being, play‐based learning, and delayed academic instruction (Sahlberg 2011). The moderate development from 2 to 3 dimensions may reflect this philosophy—skill differentiation proceeds steadily but without the acceleration seen in more academically competitive contexts like Suzhou. This interpretation aligns with research suggesting that different educational philosophies may produce different developmental trajectories in social–emotional organization (Jones and Doolittle 2017).

The exceptional cases: Ottawa and Bogota/Moscow. Ottawa's four‐dimension structure at age 15, with Collaboration as an independent dimension, is notable. Canada's official multiculturalism policy and emphasis on inclusive education (Kymlicka 1995) may foster earlier differentiation of interpersonal skills. Bogota and Moscow's four‐dimension structures at age 15, with different configurations, suggest that multiple pathways to skill organization exist—there may be no single “correct” structure, but rather culturally adaptive organizations that support effective functioning in specific contexts.

The observed cross‐city variation can be interpreted by Hofstede's cultural dimension theory (2001), such as the individualism–collectivism division. For instance, individualistic contexts (e.g., Houston, Ottawa) emphasize personal autonomy, promoting earlier differentiation of self‐oriented skills like Task Performance (Heine et al. 1999). Collectivist contexts (e.g., Suzhou, Daegu) prioritize group harmony, leading to greater integration of relational skills (Li 2012). Cross‐cultural evidence shows that categorization strategies vary systematically across cultures (Unsworth et al. 2005); East Asian children organize objects thematically while Western children emphasize taxonomic organization (Ji et al. 2004). The failure to replicate the five‐dimension structure uniformly may reflect culturally adaptive variations rather than measurement flaws (Kagitcibasi 2007). However, alternative explanations—including translation equivalence (Tsai et al. 2026), response styles (e.g., modesty bias in East Asian respondents vs. extreme responding in Western respondents) (Guo and Spina 2019), measurement interpretation (Van de Vijver and Leung 1997), school environment factors (e.g., intensity of SEL implementation) (DiPerna et al. 2024), and social desirability (Randall et al. 1993)—must also be considered before drawing strong cultural conclusions. In addition, cultural models of the self (Markus and Kitayama 1991) suggest that psychological constructs may not be organized equivalently across societies, further complicating direct structural comparisons. These interpretations are speculative and require direct measurement of cultural values to test rigorously.

### 4.3 Near–Far Continuum: Dimensional Ordering and Inter‐Distance

A unique contribution of the current study is confirming the near–far continuum of Task Performance—Emotion Regulation—Collaboration—Open‐Mindedness—Engaging with Others based on their distance between one another, which is in line with the SSES framework based on the Big Five theory (Goldberg 1993). That is to say, the inward‐oriented characteristics of Task Performance (conscientiousness), in terms of Responsibility, Self‐Control, and Persistence, are far away from the outward‐oriented characteristics of Engaging with Others (extraversion), in terms of Sociability, Assertiveness, and Energy.

The variation of combination and independence of these dimensions and their skill facets across cities in each age group provides clues for better understanding of social–emotional skills and development in each context. Specifically, the Task Performance dimension could be identified in six of the ten contexts for the 15‐year‐olds, including Daegu, Houston, Istanbul, Moscow, Suzhou, and Ottawa, suggesting that this dimension matures in many contexts during early adolescence. Our result is consistent with the findings of Mõttus et al. (2012), who used anchoring vignettes to test whether people from 21 countries have different standards for conscientiousness of the Big Five. Additionally, Emotion Regulation appeared as an independent dimension in Suzhou. Collaboration appeared as an independent dimension in Sintra. Engaging with Others and Open‐Mindedness appeared as two independent dimensions in Ottawa. The emergence of these independent dimensions suggested their maturity, and perhaps their importance, in the school lives of the 15‐year‐olds in Suzhou, Sintra, and Ottawa respectively.

### 4.4 Theoretical Reflection: Network Communities and Latent Factors

A critical theoretical distinction should be noted: the “communities” identified through EGA are not equivalent to the “latent factors” estimated in traditional factor analysis. In network psychometrics, a community refers to a cluster of variables that are directly related to one another after conditioning on all other variables in the network, reflecting patterns of conditional dependence. In contrast, a latent factor is a common cause hypothesized to explain the correlations among a set of indicators under the assumption of local independence, i.e., no residual correlations among indicators after accounting for the factor. These two constructs are neither mathematically nor conceptually identical.

Accordingly, the finding that the SSES skill facets do not coalesce into five distinct communities does not automatically invalidate the five‐factor model. Rather, it suggests that the pattern of conditional dependencies among the items is inconsistent with a simple structure where items within a factor are conditionally independent given the latent variable—a violation of the local independence assumption. In other words, the skills may share common causes (the latent factors) while also having direct relationships with each other beyond those explained by the factors, a situation that would produce network communities deviating from the factor structure (van Bork et al. 2021).

Thus, our findings should be interpreted as evidence about the pattern of conditional dependencies among skill facets, not as a direct test of the existence of latent factors. The observed age and cultural variation suggest that the organization of direct relationships among skills changes with development and across contexts. This has practical implications: if skills directly influence each other rather than merely being outcomes of common causes, interventions targeting one skill may have spillover effects on others—effects that could differ by age and culture. Future research should consider both latent variable and network approaches to provide a more complete picture of skill organization.

### 4.5 Implications for Policy and Practice

For assessment framework developers. The failure of the five‐dimension structure to emerge in any participating city, particularly among 10‐year‐olds, cautions against the uncritical application of adult‐derived personality models to child and adolescent populations, at least under the current measurement operationalization. Assessment frameworks intended for cross‐national use should undergo rigorous developmental validation before implementation. For younger age groups (e.g., 10‐year‐olds), where skills remain largely undifferentiated, instruments with simplified structures or fewer dimensions may be more developmentally appropriate. To ensure cross‐age comparability while respecting cognitive differences, separate yet linked systems for 10‐ and 15‐year‐olds with anchor items should be considered. More fundamentally, the cross‐city variation in dimensional configurations at age 15 suggests that the assumption of structural universality requires empirical scrutiny rather than theoretical presumption. The skill domain Collaboration merges with Emotion Regulation in Suzhou, Daegu and Houston but emerges independently in Ottawa among 15‐year‐olds, direct cross‐city comparisons on a standalone dimension are invalid. In contexts where Collaboration merges with Emotion Regulation, the construct may reflect relational harmony maintenance rather than cooperative teamwork. Future iterations of large‐scale assessments might consider adaptive frameworks that preserve core comparability while allowing for culturally‐grounded dimensional variations, or employ hybrid approaches combining etic (universal) and emic (culture‐specific) components.

For educational policymakers. The divergent developmental trajectories observed across cities, specifically Suzhou's rapid progression to four dimensions versus Houston, Sintra, and Istanbul's early‐but‐stagnant differentiation, suggest that national and local policies shape not only the level of skill development but also its very organization. Policymakers should recognize that social–emotional learning (SEL) mandates may accelerate early differentiation but do not guarantee continued progression; the content and emphasis of SEL curricula likely matter as much as their presence. The finding that 10‐year‐olds in most cities show undifferentiated structures suggests that primary education should focus on foundational social–emotional experiences rather than expecting fine‐grained skill distinctions to emerge. For early adolescents, curricula might be designed to support progressive differentiation while remaining attentive to local cultural configurations. For instance, contexts where Collaboration remains integrated with other dimensions may benefit from different pedagogical approaches than contexts where it emerges independently.

For school leaders and teachers. The network perspective validated in this study, wherein skills exhibit direct interrelationships rather than merely reflecting common latent causes, offers a practical framework for intervention design. If skills influence one another directly, then targeted interventions may produce spillover effects throughout the skill network. For instance, a program strengthening Persistence (a Task Performance facet) might, through network connections, also enhance aspects of Emotion Regulation or Collaboration without directly targeting them. Educators might therefore design integrated activities that leverage these interconnections rather than addressing skills in isolation.

### 4.6 Limitations

When interpreting the results of this study, several limitations must be acknowledged. First, the network analysis itself relies on correlations, which inherently limits causal inference; the observed relationships cannot confirm directional or causative effects underlying social–emotional skills differences. Second, this study compared network structures of the social–emotional skills measurement model across sub‐samples from ten cities, that is, the configural invariance rather than deeper level invariance. Third, while the dimensional structures were consistent across most groups, a few exceptions (e.g., Manizales10, Helsin10) showed inconsistency, warranting cautious interpretation and future investigation into the sources of this instability. Future studies should adopt more complex designs, such as longitudinal or experimental frameworks, to explore causality and strengthen cross‐cultural validity through expanded sampling and advanced invariance‐testing approaches.

## 5 Conclusion

The OECD SSES presents a unique opportunity to perform a cross‐contextual analysis of social–emotional skills among 10‐year‐olds and 15‐year‐olds. This study suggests that Task Performance may serve as a stable dimension of social–emotional skills among early adolescents in many contexts. From childhood to early adolescence, students' social–emotional skills not only show stronger connections among skill facets (Huo and Ning 2025) but also distinct functional divisions. There are cultural differences in the dimensions of social–emotional skills, such as the relative maturation of the 10‐year‐olds in Houston, Sintra, and Istanbul, as well as the significant development of the 15‐year‐olds in Suzhou. This study informs researchers, policy‐makers, and practitioners of the limitations of the Big‐Five based SSES functional zoning framework considering the immaturity of children and early adolescents. While the observed inter‐city variations in dimensional configuration, ranging from tight integration to full autonomy across social–emotional skill facets within each age cohort, offer critical insights for contextualizing competency development patterns within distinct sociocultural ecosystems.

## Funding

This work was supported by the 2024 Shuguang Research Project entitled Research on Pathways for China‐Europe Basic Education Exchange and Cooperation (24SG43), which was funded by Shanghai Municipal Education Commission and Shanghai Education Development Foundation.

## Ethics Statement

This study utilizes data from the 2019 OECD Survey on Social and Emotional Skills. The OECD is committed to ethical data collection practices, and this data was collected with full consent of the participants that ensured its anonymity and confidentiality.

## Conflicts of Interest

The authors declare no conflicts of interest.

## Supporting information


**Table S1:** Robustness of EGA solutions: Parameter sensitivity analysis and bootstrap stability (descriptive statistics) across cities.
**Table S2:** Goodness‐of‐fit indicators of hierarchical CFA models across cities.
**Table S3:** First‐order (skill to domain) and second‐order (domain to general factor) loadings across cities based on hierarchical CFA model.
**Table S4:** Goodness‐of‐fit indicators of bifactor CFA models across cities.
**Table S5:** Domain‐specific and general factor loadings across cities based on bifactor CFA model.

## Data Availability

The data used in this study are publicly available and can be accessed through the OECD website. The OECD SSES Round 1 Database can be found at https://www.oecd.org/en/data/datasets/SSES‐Round‐1‐Database.html.
