# Supplementary material for: Exploratory Graph Analysis Failed to Reproduce the Hypothesized Functional Zoning Framework in the OECD Survey on Social and Emotional Skills
Source: Psych J. 2026 Jun 17;15(3):e70108. doi: 10.1002/pchj.70108 (PMC13273016; doi:10.1002/pchj.70108)
Supplement: Supplementary file 1 — Table S1: Robustness of EGA solutions: Parameter sensitivity analysis and bootstrap stability (descriptive statistics) across cities. Table S2: Goodness‐of‐fit indicators of hierarchical CFA models across cities. Table S3: First‐order (skill to domain) and second‐order (domain to general factor) loadings across cities based on hierarchical CFA model. Table S4: Goodness‐of‐fit indicators of bifactor CFA models across cities. Table S5: Domain‐specific and general factor loadings across cities based on bifactor CFA model. [file PCHJ-15-e70108-s001.docx]

**Supplementary Materials**

**Robustness Results**

**Table S1**. *Robustness of EGA solutions: Parameter sensitivity analysis and bootstrap stability (descriptive statistics) across cities*

| City | Age | Original_dim | Parameter sensitivity (γ) | | | | | Bootstrap stability | | | | | |
| --- | --- | --- | --- | --- | --- | --- | --- | --- | --- | --- | --- | --- | --- |
|  |  |  | γ = 0 | γ = 0.25 | γ = 0.5 | γ = 0.75 | γ = 1 | n.Boots | median.dim | SE.dim | CI.dim | Lower.CI | Upper.CI |
| Suzhou | 10 | 1 | 1 | 1 | 1 | 1 | 1 | 1000 | 1 | 0 | 0 | 1 | 1 |
|  | 15 | 4 | 1 | 4 | 4 | 1 | 4 |  | 4 | 0.824 | 1.617 | 2.383 | 5.617 |
| Houston | 10 | 3 | 3 | 3 | 3 | 3 | 3 |  | 3 | 0.371 | 0.729 | 2.271 | 3.729 |
|  | 15 | 3 | 3 | 3 | 3 | 3 | 3 |  | 3 | 0.071 | 0.138 | 2.862 | 3.138 |
| Ottawa | 10 | 2 | 2 | 2 | 2 | 2 | 2 |  | 2 | 0.602 | 1.181 | 0.819 | 3.181 |
|  | 15 | 4 | 4 | 4 | 4 | 4 | 4 |  | 3 | 0.339 | 0.665 | 2.335 | 3.665 |
| Bogota | 10 | 2 | 2 | 2 | 2 | 2 | 2 |  | 2 | 0.095 | 0.186 | 1.814 | 2.186 |
|  | 15 | 4 | 4 | 4 | 4 | 4 | 4 |  | 3 | 0.341 | 0.668 | 2.332 | 3.668 |
| Manizales | 10 | 1 | 1 | 2 | 2 | 2 | 2 |  | 2 | 0.351 | 0.689 | 1.311 | 2.689 |
|  | 15 | 3 | 3 | 3 | 3 | 3 | 3 |  | 3 | 0.453 | 0.888 | 2.112 | 3.888 |
| Helsinki | 10 | 2 | 2 | 2 | 2 | 2 | 2 |  | 3 | 1.051 | 2.063 | 0.937 | 5.063 |
|  | 15 | 3 | 3 | 3 | 3 | 3 | 3 |  | 3 | 0.330 | 0.647 | 2.353 | 3.647 |
| Moscow | 10 | 2 | 2 | 2 | 2 | 2 | 2 |  | 2 | 0.648 | 1.272 | 0.728 | 3.272 |
|  | 15 | 4 | 4 | 4 | 4 | 4 | 4 |  | 4 | 0.274 | 0.537 | 3.463 | 4.537 |
| Istanbul | 10 | 3 | 3 | 3 | 3 | 3 | 3 |  | 3 | 0.623 | 1.222 | 1.778 | 4.222 |
|  | 15 | 3 | 3 | 3 | 3 | 3 | 3 |  | 3 | 0.458 | 0.898 | 2.102 | 3.898 |
| Daegu | 10 | 1 | 1 | 1 | 1 | 1 | 1 |  | 1 | 0 | 0 | 1 | 1 |
|  | 15 | 3 | 3 | 3 | 3 | 3 | 3 |  | 3 | 0.055 | 0.107 | 2.893 | 3.107 |
| Sintra | 10 | 3 | 3 | 3 | 3 | 3 | 3 |  | 3 | 0.556 | 1.092 | 1.908 | 4.092 |
|  | 15 | 3 | 4 | 3 | 3 | 3 | 3 |  | 3 | 0.771 | 1.512 | 1.488 | 4.512 |

**Hierarchical and Bifactor CFA Results**

To assess the potential hierarchical structure of the OECD model and to test whether the dimensions identified by EGA are method-specific, we used skill-level scores as indicators for each domain and conducted hierarchical confirmatory factor analysis (CFA) and bifactor CFA, respectively (see also Huo & Ning, 2025b*,* for the OECD SSES 2019 data). These analyses were performed separately for each of the 20 city-age groups (10 cities × two age groups: 10 years and 15 years). Each model fit was examined using the lavaan package (Rosseel, 2012). Model fit was assessed by comparing the Comparative Fit Index (CFI), Tucker-Levy Index (TLI), Root Mean Square Error of Approximation (RMSEA), and Standardized Root Mean Square Residual (SRMR). Criteria for good model fit include CFI and TLI values above 0.95, an RMSEA value below 0.05, and an SRMR value below 0.08 (Kline, 2016).

Both the hierarchical and bifactor CFA models consistently showed poor fit across the 20 city‑age groups. For both the hierarchical CFA (Table S2) and the bifactor CFA (Table S4), only one group (Daegu’s 10-year-old cohort) achieved a CFI value greater than 0.95, while all RMSEA values exceeded 0.05. Additional results, including factor loadings, are presented in Tables S2–S5. These results imply that the five‑domain framework of the OECD may not be an accurate categorization of the 15 skill facets into five domains, regardless of whether a hierarchical or a bifactor structure is assumed.

**References**

Huo, M., & Ning, B. (2025b). Mapping the maze: A network analysis of social-emotional skills among children and adolescents with social-emotional difficulties. *British Journal of Psychology, 116*(1), 233-249. https://doi.org/10.1111/bjop.12751

Kline, R. B. (2016). *Principles and Practice of Structural Equation Modeling*. Guilford Press.

Rosseel, Y. (2012). lavaan: An R Package for Structural Equation Modeling. *Journal of Statistical Software, 48*(2), 1–36. https://doi.org/10.18637/jss.v048.i02

**Table S2**. *Goodness-of-fit indicators of hierarchical CFA models across cities*

| City | Age | χ2 | df | CFI | TLI | RMSEA | SRMR | AIC | BIC |
| --- | --- | --- | --- | --- | --- | --- | --- | --- | --- |
| Suzhou | 10 | 2130.345^***^ | 85 | 0.942 | 0.928 | 0.081 | 0.038 | 632087.997 | 632304.901 |
|  | 15 | 3526.595^***^ |  | 0.895 | 0.87 | 0.106 | 0.054 | 602377.55 | 602594.261 |
| Houston | 10 | 2482.225^***^ |  | 0.881 | 0.853 | 0.092 | 0.058 | 575688.84 | 575902.483 |
|  | 15 | 3849.016^***^ |  | 0.821 | 0.779 | 0.12 | 0.078 | 531462.578 | 531673.858 |
| Ottawa | 10 | 2668.857^***^ |  | 0.874 | 0.845 | 0.098 | 0.059 | 546441.693 | 546653.723 |
|  | 15 | 2676.229^***^ |  | 0.792 | 0.743 | 0.118 | 0.087 | 373337.133 | 373536.069 |
| Bogota | 10 | 1869.111^***^ |  | 0.907 | 0.885 | 0.079 | 0.05 | 588876.725 | 589091.329 |
|  | 15 | 3860.889^***^ |  | 0.796 | 0.747 | 0.115 | 0.082 | 572467.131 | 572681.258 |
| Manizales | 10 | 1741.505^***^ |  | 0.913 | 0.892 | 0.078 | 0.048 | 556544.383 | 556757.061 |
|  | 15 | 4082.131^***^ |  | 0.798 | 0.751 | 0.115 | 0.084 | 603492.559 | 603708.486 |
| Helsinki | 10 | 2288.391^***^ |  | 0.889 | 0.863 | 0.093 | 0.055 | 507829.146 | 508039.275 |
|  | 15 | 3545.167^***^ |  | 0.776 | 0.724 | 0.129 | 0.089 | 414485.217 | 414688.107 |
| Moscow | 10 | 2554.692^***^ |  | 0.896 | 0.871 | 0.093 | 0.054 | 575443.857 | 575657.795 |
|  | 15 | 4219.907^***^ |  | 0.809 | 0.765 | 0.119 | 0.079 | 587363.921 | 587578.699 |
| Istanbul | 10 | 1930.021^***^ |  | 0.908 | 0.886 | 0.09 | 0.051 | 474708.028 | 474914.524 |
|  | 15 | 3597.333^***^ |  | 0.809 | 0.764 | 0.114 | 0.077 | 553643.983 | 553856.091 |
| Daegu | 10 | 1406.315^***^ |  | 0.955 | 0.944 | 0.072 | 0.033 | 517171.429 | 517381.722 |
|  | 15 | 3849.016^***^ |  | 0.821 | 0.779 | 0.12 | 0.078 | 531462.578 | 531673.858 |
| Sintra | 10 | 1812.378^***^ |  | 0.861 | 0.828 | 0.096 | 0.065 | 379101.838 | 379301.062 |
|  | 15 | 2032.314^***^ |  | 0.761 | 0.705 | 0.118 | 0.096 | 281646.266 | 281835.202 |

*Note.* $**p<.01. ***p<.001$. CFA ≥0.95 indicates good fit; TLI ≥0.95 indicates good fit; RMSEA <0.05 indicates good fit. SRMR <0.08 indicates good fit.

**Table S3**. *First-order (skill to domain) and second-order (domain to general factor) loadings across cities based on hierarchical CFA model*

| Domain | Skill | Suzhou | | | | Houston | | | | Ottawa | | | | Bogota | | | | Manizales | | | |
| --- | --- | --- | --- | --- | --- | --- | --- | --- | --- | --- | --- | --- | --- | --- | --- | --- | --- | --- | --- | --- | --- |
|  |  | 10-year-olds | | 15-year-olds | | 10-year-olds | | 15-year-olds | | 10-year-olds | | 15-year-olds | | 10-year-olds | | 15-year-olds | | 10-year-olds | | 15-year-olds | |
|  |  | First | Second | First | Second | First | Second | First | Second | First | Second | First | Second | First | Second | First | Second | First | Second | First | Second |
| Collaboration | EMP | 0.854 | 0.95 | 0.829 | 0.91 | 0.58 | 0.932 | 0.707 | 0.877 | 0.738 | 0.939 | 0.694 | 0.823 | 0.593 | 0.964 | 0.655 | 0.816 | 0.615 | 0.931 | 0.679 | 0.786 |
|  | TRU | 0.607 |  | 0.697 |  | 0.56 |  | 0.451 |  | 0.577 |  | 0.454 |  | 0.524 |  | 0.427 |  | 0.53 |  | 0.391 |  |
|  | COO | 0.888 |  | 0.9 |  | 0.865 |  | 0.888 |  | 0.859 |  | 0.831 |  | 0.834 |  | 0.852 |  | 0.856 |  | 0.842 |  |
| Emotional Regulation | STR | 0.687 | 0.918 | 0.691 | 0.852 | 0.477 | 0.87 | 0.583 | 0.778 | 0.57 | 0.863 | 0.611 | 0.73 | 0.378 | 0.922 | 0.501 | 0.784 | 0.377 | 0.922 | 0.538 | 0.809 |
|  | OPT | 0.811 |  | 0.854 |  | 0.8 |  | 0.833 |  | 0.805 |  | 0.827 |  | 0.775 |  | 0.823 |  | 0.782 |  | 0.816 |  |
|  | EMO | 0.775 |  | 0.754 |  | 0.671 |  | 0.618 |  | 0.732 |  | 0.62 |  | 0.607 |  | 0.604 |  | 0.606 |  | 0.6 |  |
| Engaging With Others | ASS | 0.504 | 0.947 | 0.493 | 0.908 | 0.46 | 0.799 | 0.545 | 0.846 | 0.399 | 0.798 | 0.557 | 0.809 | 0.269 | 0.865 | 0.449 | 0.855 | 0.253 | 0.868 | 0.459 | 0.902 |
|  | SOC | 0.736 |  | 0.769 |  | 0.719 |  | 0.713 |  | 0.778 |  | 0.709 |  | 0.673 |  | 0.674 |  | 0.716 |  | 0.62 |  |
|  | ENE | 0.759 |  | 0.806 |  | 0.624 |  | 0.733 |  | 0.541 |  | 0.694 |  | 0.692 |  | 0.775 |  | 0.704 |  | 0.758 |  |
| Open-Mindedness | CUR | 0.829 | 0.953 | 0.828 | 0.879 | 0.744 | 0.908 | 0.757 | 0.865 | 0.748 | 0.856 | 0.723 | 0.792 | 0.734 | 0.914 | 0.717 | 0.784 | 0.748 | 0.913 | 0.709 | 0.778 |
|  | TOL | 0.743 |  | 0.689 |  | 0.605 |  | 0.665 |  | 0.585 |  | 0.541 |  | 0.627 |  | 0.601 |  | 0.636 |  | 0.57 |  |
|  | CRE | 0.783 |  | 0.749 |  | 0.655 |  | 0.71 |  | 0.638 |  | 0.626 |  | 0.706 |  | 0.693 |  | 0.728 |  | 0.761 |  |
| Task Performance | RES | 0.824 | 0.915 | 0.822 | 0.84 | 0.783 | 0.903 | 0.804 | 0.796 | 0.807 | 0.884 | 0.843 | 0.722 | 0.766 | 0.855 | 0.818 | 0.681 | 0.755 | 0.886 | 0.81 | 0.658 |
|  | SEL | 0.754 |  | 0.662 |  | 0.734 |  | 0.675 |  | 0.749 |  | 0.65 |  | 0.725 |  | 0.602 |  | 0.718 |  | 0.577 |  |
|  | PER | 0.87 |  | 0.889 |  | 0.777 |  | 0.828 |  | 0.755 |  | 0.803 |  | 0.749 |  | 0.793 |  | 0.772 |  | 0.853 |  |

Table S3 (continued)

**Table S3**. (Continued)

| Domain | Skill | Helsinki | | | | Moscow | | | | Istanbul | | | | Daegu | | | | Sintra | | | |
| --- | --- | --- | --- | --- | --- | --- | --- | --- | --- | --- | --- | --- | --- | --- | --- | --- | --- | --- | --- | --- | --- |
|  |  | 10-year-olds | | 15-year-olds | | 10-year-olds | | 15-year-olds | | 10-year-olds | | 15-year-olds | | 10-year-olds | | 15-year-olds | | 10-year-olds | | 15-year-olds | |
|  |  | First | Second | First | Second | First | Second | First | Second | First | Second | First | Second | First | Second | First | Second | First | Second | First | Second |
| Collaboration | EMP | 0.734 | 0.957 | 0.73 | 0.797 | 0.714 | 0.906 | 0.697 | 0.791 | 0.666 | 0.975 | 0.696 | 0.812 | 0.869 | 0.944 | 0.707 | 0.877 | 0.651 | 0.955 | 0.64 | 0.816 |
|  | TRU | 0.66 |  | 0.566 |  | 0.636 |  | 0.563 |  | 0.443 |  | 0.335 |  | 0.606 |  | 0.451 |  | 0.598 |  | 0.397 |  |
|  | COO | 0.852 |  | 0.839 |  | 0.894 |  | 0.909 |  | 0.848 |  | 0.827 |  | 0.883 |  | 0.888 |  | 0.853 |  | 0.885 |  |
| Emotional Regulation | STR | 0.521 | 0.866 | 0.565 | 0.811 | 0.657 | 0.881 | 0.733 | 0.747 | 0.626 | 0.848 | 0.62 | 0.751 | 0.707 | 0.917 | 0.583 | 0.778 | 0.306 | 0.834 | 0.528 | 0.608 |
|  | OPT | 0.837 |  | 0.887 |  | 0.795 |  | 0.757 |  | 0.827 |  | 0.753 |  | 0.804 |  | 0.833 |  | 0.76 |  | 0.693 |  |
|  | EMO | 0.674 |  | 0.547 |  | 0.76 |  | 0.699 |  | 0.729 |  | 0.66 |  | 0.797 |  | 0.618 |  | 0.622 |  | 0.69 |  |
| Engaging With Others | ASS | -0.024 | 0.907 | 0.413 | 0.844 | 0.157 | 0.868 | 0.442 | 0.854 | 0.127 | 0.921 | 0.418 | 0.806 | 0.639 | 0.872 | 0.545 | 0.846 | 0.077 | 0.765 | 0.286 | 0.657 |
|  | SOC | 0.761 |  | 0.663 |  | 0.697 |  | 0.712 |  | 0.727 |  | 0.651 |  | 0.793 |  | 0.713 |  | 0.775 |  | 0.662 |  |
|  | ENE | 0.593 |  | 0.813 |  | 0.621 |  | 0.772 |  | 0.749 |  | 0.831 |  | 0.777 |  | 0.733 |  | 0.619 |  | 0.777 |  |
| Open-Mindedness | CUR | 0.784 | 0.908 | 0.753 | 0.836 | 0.733 | 0.925 | 0.684 | 0.789 | 0.794 | 0.93 | 0.773 | 0.769 | 0.84 | 0.912 | 0.757 | 0.865 | 0.732 | 0.9 | 0.781 | 0.789 |
|  | TOL | 0.622 |  | 0.507 |  | 0.646 |  | 0.581 |  | 0.583 |  | 0.511 |  | 0.716 |  | 0.665 |  | 0.647 |  | 0.639 |  |
|  | CRE | 0.643 |  | 0.652 |  | 0.639 |  | 0.647 |  | 0.697 |  | 0.715 |  | 0.775 |  | 0.71 |  | 0.645 |  | 0.573 |  |
| Task Performance | RES | 0.772 | 0.875 | 0.814 | 0.752 | 0.839 | 0.855 | 0.844 | 0.74 | 0.83 | 0.907 | 0.807 | 0.805 | 0.851 | 0.939 | 0.804 | 0.796 | 0.766 | 0.857 | 0.775 | 0.782 |
|  | SEL | 0.709 |  | 0.616 |  | 0.805 |  | 0.681 |  | 0.808 |  | 0.68 |  | 0.795 |  | 0.675 |  | 0.716 |  | 0.565 |  |
|  | PER | 0.763 |  | 0.821 |  | 0.782 |  | 0.779 |  | 0.813 |  | 0.817 |  | 0.822 |  | 0.828 |  | 0.78 |  | 0.803 |  |

**Table S4**. *Goodness-of-fit indicators of bifactor CFA models across cities*

| City | Age | χ2 | df | CFI | TLI | RMSEA | SRMR | AIC | BIC |
| --- | --- | --- | --- | --- | --- | --- | --- | --- | --- |
| Suzhou | 10 | 1585.777^***^ | 70 | 0.957 | 0.935 | 0.077 | 0.033 | 631573.430 | 631883.293 |
|  | 15 | 3196.540^***^ |  | 0.905 | 0.857 | 0.111 | 0.053 | 602077.495 | 602387.082 |
| Houston | 10 | 1611.318^***^ |  | 0.923 | 0.885 | 0.082 | 0.045 | 574847.933 | 575153.138 |
|  | 15 | 2435.560^***^ |  | 0.887 | 0.831 | 0.105 | 0.057 | 530079.122 | 530380.951 |
| Ottawa | 10 | 2270.542^***^ |  | 0.893 | 0.839 | 0.100 | 0.054 | 546073.379 | 546376.279 |
|  | 15 | 1561.525^***^ |  | 0.880 | 0.821 | 0.099 | 0.058 | 372252.430 | 372536.623 |
| Bogota | 10 | 1081.153^***^ |  | 0.947 | 0.921 | 0.065 | 0.034 | 588118.768 | 588425.344 |
|  | 15 | 3375.433^***^ |  | 0.821 | 0.731 | 0.119 | 0.078 | 572011.674 | 572317.570 |
| Manizales | 10 | 1869.299^***^ |  | 0.905 | 0.858 | 0.089 | 0.048 | 556702.177 | 557006.003 |
|  | 15 | 2670.751^***^ |  | 0.869 | 0.803 | 0.103 | 0.062 | 602111.179 | 602419.645 |
| Helsinki | 10 | 1400.652^***^ |  | 0.933 | 0.900 | 0.080 | 0.040 | 506971.406 | 507271.591 |
|  | 15 | 2153.050^***^ |  | 0.865 | 0.798 | 0.111 | 0.065 | 413123.100 | 413412.944 |
| Moscow | 10 | 1444.768^***^ |  | 0.942 | 0.913 | 0.077 | 0.039 | 574363.933 | 574669.559 |
|  | 15 | 2786.939^***^ |  | 0.875 | 0.812 | 0.107 | 0.065 | 585960.952 | 586267.778 |
| Istanbul | 10 | 1916.699^***^ |  | 0.908 | 0.862 | 0.099 | 0.049 | 474724.706 | 475019.700 |
|  | 15 | 2775.720^***^ |  | 0.853 | 0.779 | 0.110 | 0.069 | 552852.371 | 553155.382 |
| Daegu | 10 | 1056.469^***^ |  | 0.966 | 0.949 | 0.068 | 0.028 | 516851.583 | 517152.001 |
|  | 15 | 2435.560^***^ |  | 0.887 | 0.831 | 0.105 | 0.057 | 530079.122 | 530380.951 |
| Sintra | 10 | 1110.828^***^ |  | 0.916 | 0.874 | 0.082 | 0.045 | 378430.289 | 378714.894 |
|  | 15 | 1427.800^***^ |  | 0.834 | 0.750 | 0.109 | 0.078 | 281071.752 | 281341.660 |

*Note.* $**p<.01. ***p<.001$. CFA ≥0.95 indicates good fit; TLI ≥0.95 indicates good fit; RMSEA <0.05 indicates good fit. SRMR <0.08 indicates good fit.

**Table S5**. *Domain-specific and general factor loadings across cities based on bifactor CFA model*

| Domain | Skill | Suzhou | | | | Houston | | | | Ottawa | | | | Bogota | | | | Manizales | | | |
| --- | --- | --- | --- | --- | --- | --- | --- | --- | --- | --- | --- | --- | --- | --- | --- | --- | --- | --- | --- | --- | --- |
|  |  | 10-year-olds | | 15-year-olds | | 10-year-olds | | 15-year-olds | | 10-year-olds | | 15-year-olds | | 10-year-olds | | 15-year-olds | | 10-year-olds | | 15-year-olds | |
|  |  | Specific | General | Specific | General | Specific | General | Specific | General | Specific | General | Specific | General | Specific | General | Specific | General | Specific | General | Specific | General |
| Collaboration | COO | 0.385 | 0.664 | 0.000 | 0.885 | 0.298 | 0.831 | -0.280 | 0.825 | 0.108 | 0.800 | 0.389 | 0.733 | 0.696 | 0.775 | 0.282 | 0.655 | -0.355 | 5.541 | 0.536 | 0.597 |
|  | EMP | 0.359 | 0.648 | 0.000 | 0.823 | 0.085 | 0.597 | -0.297 | 0.659 | 1.059 | 0.676 | 0.398 | 0.603 | 0.067 | 0.580 | 0.948 | 0.456 | -0.255 | 3.985 | 0.629 | 0.432 |
|  | TRU | 0.257 | 0.458 | 0.000 | 0.702 | 0.327 | 0.500 | 0.016 | 0.450 | -0.036 | 0.604 | -0.084 | 0.482 | 0.046 | 0.510 | 0.053 | 0.429 | -0.224 | 3.495 | 0.077 | 0.421 |
| Emotional Regulation | EMO | 0.270 | 0.705 | 0.432 | 0.623 | 10.890 | -10.591 | 9.857 | -9.258 | 0.177 | 0.668 | 11.144 | -10.554 | 0.590 | 0.485 | 0.683 | 0.516 | 0.015 | -9.330 | 0.240 | 0.559 |
|  | OPT | 0.223 | 0.753 | 0.423 | 0.714 | 10.316 | -9.876 | 27.233 | -26.487 | 0.158 | 0.737 | 28.389 | -27.637 | 0.140 | 0.720 | 0.149 | 0.736 | -0.008 | 5.213 | 0.071 | 0.775 |
|  | STR | 0.475 | 0.601 | 0.591 | 0.496 | 11.732 | -11.691 | 33.564 | -33.088 | 1.058 | 0.428 | 33.429 | -32.941 | 0.401 | 0.279 | 0.375 | 0.384 | -0.004 | 2.777 | 0.948 | 0.458 |
| Engaging With Others | ASS | -0.135 | 0.358 | 0.389 | 0.393 | 0.264 | 0.386 | 0.242 | 0.430 | 0.164 | 0.288 | 0.355 | 0.367 | 0.217 | 0.235 | 14.816 | 15.121 | -0.001 | 1.274 | 1.234 | 0.343 |
|  | ENE | 0.088 | 0.821 | 0.425 | 0.688 | 0.427 | 0.497 | 0.710 | 0.530 | 1.108 | 0.402 | 0.675 | 0.462 | 0.201 | 0.635 | 1.171 | 1.892 | -0.003 | 4.287 | 0.061 | 0.730 |
|  | SOC | 0.336 | 1.040 | 0.252 | 0.734 | 0.427 | 0.585 | 0.346 | 0.572 | 0.139 | 0.644 | 0.389 | 0.529 | 0.318 | 0.630 | 1.507 | 2.110 | -0.003 | 4.300 | 0.093 | 0.584 |
| Open-Mindedness | CRE | 0.109 | 0.768 | 9.929 | -9.344 | 0.223 | 0.631 | 0.263 | 0.616 | 23.088 | -22.577 | 0.265 | 0.517 | 0.181 | 0.665 | 0.275 | 0.559 | -0.002 | 4.716 | 15.816 | -15.287 |
|  | CUR | 0.139 | 0.798 | 10.944 | -10.296 | 0.234 | 0.711 | 0.366 | 0.656 | 3.728 | -3.094 | 0.461 | 0.570 | 0.351 | 0.673 | 0.398 | 0.492 | -0.002 | 4.771 | 19.987 | -19.559 |
|  | TOL | 0.608 | 0.722 | 7.488 | -6.924 | 0.150 | 0.603 | 0.401 | 0.588 | 3.233 | -2.739 | 0.385 | 0.437 | 0.276 | 0.568 | 0.865 | 0.314 | -0.001 | 4.051 | 18.853 | -18.594 |
| Task Performance | PER | 0.460 | 1.077 | 0.570 | 0.709 | 0.329 | 0.716 | 0.534 | 0.622 | 0.324 | 0.653 | 0.521 | 0.574 | 13.230 | 13.839 | 0.659 | 0.477 | -0.004 | 4.969 | 0.764 | 0.476 |
|  | RES | 0.397 | 1.001 | 0.439 | 0.693 | 0.349 | 0.714 | 0.575 | 0.600 | 0.584 | 0.679 | 0.645 | 0.596 | 16.338 | 16.930 | 0.633 | 0.514 | -0.004 | 4.769 | 0.646 | 0.453 |
|  | SEL | 0.429 | 0.953 | 0.449 | 0.512 | 0.436 | 0.645 | 0.294 | 0.607 | 0.257 | 0.686 | 0.364 | 0.537 | 21.449 | 22.009 | 0.353 | 0.484 | -0.004 | 5.204 | 0.336 | 0.498 |

Table S5 (continued)

**Table S5**. (continued)

| Domain | Skill | Helsinki | | | | Moscow | | | | Istanbul | | | | Daegu | | | | Sintra | | | |
| --- | --- | --- | --- | --- | --- | --- | --- | --- | --- | --- | --- | --- | --- | --- | --- | --- | --- | --- | --- | --- | --- |
|  |  | 10-year-olds | | 15-year-olds | | 10-year-olds | | 15-year-olds | | 10-year-olds | | 15-year-olds | | 10-year-olds | | 15-year-olds | | 10-year-olds | | 15-year-olds | |
|  |  | Specific | General | Specific | General | Specific | General | Specific | General | Specific | General | Specific | General | Specific | General | Specific | General | Specific | General | Specific | General |
| Collaboration | COO | 0.197 | 0.762 | 0.491 | 0.692 | 0.000 | 0.842 | 0.241 | 0.779 | 0.039 | 0.831 | -30.029 | -29.481 | 0.002 | -0.840 | -0.280 | 0.825 | 0.233 | 0.807 | 0.361 | 0.732 |
|  | EMP | 0.424 | 0.578 | 0.446 | 0.593 | 0.000 | 0.703 | 0.564 | 0.574 | 0.025 | 0.651 | -36.574 | -36.149 | 0.002 | -0.823 | -0.297 | 0.659 | 0.129 | 0.634 | 0.568 | 0.485 |
|  | TRU | -0.124 | 0.719 | 0.149 | 0.526 | 0.000 | 0.651 | -0.148 | 0.650 | 1.379 | 0.410 | -0.924 | -0.549 | 0.002 | -0.591 | 0.016 | 0.450 | 0.351 | 0.546 | -0.148 | 0.537 |
| Emotional Regulation | EMO | 0.415 | 0.398 | -8.470 | 8.883 | 0.651 | 0.562 | 1.546 | -1.030 | 0.248 | 0.613 | 0.497 | 0.517 | 0.259 | -0.707 | 9.857 | -9.258 | 0.783 | 0.439 | 0.466 | 0.452 |
|  | OPT | 0.350 | 0.568 | 31.064 | -30.034 | 0.223 | 0.734 | 1.001 | -0.273 | 0.132 | 0.730 | 0.178 | 0.726 | 0.435 | -0.701 | 27.233 | -26.487 | 0.200 | 0.622 | 0.302 | 0.627 |
|  | STR | 0.773 | -0.024 | 41.649 | -40.938 | 0.549 | 0.450 | 2.488 | -1.979 | 0.944 | 0.503 | 0.480 | 0.493 | 0.395 | -0.596 | 33.564 | -33.088 | 0.464 | 0.121 | 0.888 | 0.144 |
| Engaging With Others | ASS | 0.343 | 0.105 | 0.315 | 0.301 | 0.420 | 0.074 | 0.390 | 0.331 | 1.206 | 0.065 | 0.227 | 0.306 | 0.277 | -0.554 | 0.242 | 0.430 | 0.257 | -0.015 | 0.356 | 0.081 |
|  | ENE | 0.382 | 0.735 | 0.561 | 0.602 | 0.422 | 0.530 | 0.540 | 0.673 | 0.073 | 0.698 | 0.257 | 0.739 | 0.371 | -0.657 | 0.710 | 0.530 | 0.385 | 0.516 | 0.505 | 0.551 |
|  | SOC | 0.357 | 0.887 | 0.402 | 0.503 | 0.151 | 0.667 | 0.338 | 0.694 | 0.071 | 0.673 | 0.749 | 0.480 | 0.382 | -0.680 | 0.346 | 0.572 | 0.432 | 0.629 | 0.423 | 0.555 |
| Open-Mindedness | CRE | 0.218 | 0.519 | 0.273 | 0.548 | 0.097 | 0.599 | 0.275 | 0.481 | 1.319 | 1.969 | 0.526 | 0.496 | 0.327 | -0.679 | 0.263 | 0.616 | 0.106 | 0.607 | 0.337 | 0.365 |
|  | CUR | 0.423 | 0.573 | 0.390 | 0.653 | 0.630 | 0.615 | 0.535 | 0.472 | 0.827 | 1.574 | 0.470 | 0.557 | 0.356 | -0.736 | 0.366 | 0.656 | 0.094 | 0.675 | 0.764 | 0.434 |
|  | TOL | 0.324 | 0.452 | 0.316 | 0.427 | 0.244 | 0.576 | 0.503 | 0.401 | 13.566 | 14.048 | 0.639 | 0.203 | 0.341 | -0.626 | 0.401 | 0.588 | 0.912 | 0.585 | 0.436 | 0.368 |
| Task Performance | PER | 0.418 | 0.859 | 0.475 | 0.641 | 15.305 | 15.839 | 0.627 | 0.554 | 0.129 | 0.749 | 0.533 | 0.600 | 7.526 | 6.583 | 0.534 | 0.622 | 10.981 | 11.685 | 7.582 | 8.007 |
|  | RES | 0.390 | 0.867 | 0.495 | 0.648 | 15.031 | 15.630 | 0.571 | 0.645 | 0.846 | 0.753 | 0.620 | 0.565 | 7.497 | 6.527 | 0.575 | 0.600 | 15.506 | 16.176 | 6.806 | 7.234 |
|  | SEL | 0.564 | 0.875 | 0.408 | 0.478 | 17.935 | 18.465 | 0.580 | 0.477 | 0.125 | 0.746 | 0.416 | 0.533 | 14.348 | 13.240 | 0.294 | 0.607 | 25.679 | 26.337 | 4.023 | 4.440 |
